# Attachment insecurity, adverse childhood experiences (ACEs), and suicidality in French residential-care adolescents: a gender-differentiated study

**DOI:** 10.1186/s13034-025-01010-3

**Published:** 2025-12-15

**Authors:** Guillaume Bronsard, Nolwenn Dissaux, Nathalie Bruneau, Issaga Diallo, Mélanie Sanchez, Laurent Boyer, Nathalie Lavenne-Collot

**Affiliations:** 1 Department of Child and Adolescent Psychiatry, Brest University Hospital, Bohars, France; 2 University of Western Brittany, Brest, France; 3 EA 7479, SPURBO, University of Western Brittany, Brest, France; 4 EA 3279, CEReSS, Aix-Marseille University, Marseille, France; 5 Public Mental Health Institution (EPSM), Guadeloupe, France; 6 Department of Medical Information, Assistance Publique, Hôpitaux de Marseille (AP-HM), Marseille, France; 7 INSERM UMR 1018, CESP, PsyDev NDTA Team, Paris-Saclay University, UVSQ, Villejuif, France; 8 Necker–Enfants Malades University Hospital, Paris, France; 9 INSERM UMR 1101, LaTIM, Brest, France

**Keywords:** Residential treatment, Suicide, Attachment behavior, Child abuse, Adolescent, Sex factors

## Abstract

**Background:**

Suicidality is alarmingly prevalent among adolescents placed in residential child welfare facilities, often as a consequence of early adverse childhood experiences (ACEs) and disrupted attachment relationships. Although these vulnerabilities are well established, the gender-specific mechanisms underlying suicidality in institutionalized youth remain poorly understood. Clarifying how trauma exposure and attachment insecurity interact with mental health symptoms is critical to inform targeted prevention.

**Methods:**

In a cross-sectional study, 98 adolescents aged 12–17 years (54 girls, 44 boys; M = 14.34, SD = 2.08) living in French residential care completed validated self-report instruments assessing ACEs, attachment security, depressive and anxiety symptoms, and suicidality. Descriptive statistics, gender comparisons, and multivariate logistic regressions were used to identify predictors of suicidality, with all predictors standardized prior to entry.

**Results:**

One-third of participants (33%) reported suicidal ideation or at least one suicide attempt. Emotional and physical abuse were the most frequent ACEs. Cumulative ACEs and attachment insecurity were independently associated with suicidality, and both correlated with heightened anxiety and depressive symptoms. Gender-stratified analyses showed that suicidality in girls was primarily linked to maternal alienation and emotional dysregulation, whereas in boys it was more strongly related to cumulative trauma exposure and depressive symptoms.

**Conclusions:**

Findings highlight suicidality as a major concern in residential care and identify two complementary risk pathways: adversity-related and attachment‐related. Trauma-informed and attachment-based approaches—supported by systematic screening and the integration of mental health professionals within child welfare systems—may enhance early detection and individualized care. While contextualized in the French system, these mechanisms likely generalize across jurisdictions, underscoring the global need for gender-sensitive, relationally focused suicide prevention.

**Supplementary Information:**

The online version contains supplementary material available at 10.1186/s13034-025-01010-3.

## Introduction

Adolescents placed in child welfare systems experience markedly higher rates of psychiatric morbidity—estimated at three to five times those observed in the general adolescent population [1, 2]. This excess burden reflects the convergence of early adversity, unstable caregiving, and limited access to consistent psychological support. Suicidality—encompassing ideation, behaviors, and attempts—remains one of the most prevalent and lethal outcomes in this group [3, 4]. Yet, despite the magnitude of the problem, the mechanisms linking early vulnerability to suicide risk—particularly those involving attachment—are still poorly understood.

Unlike the extensive literature on foster care in anglophone countries, France relies predominantly on residential placements, where adolescents are cared for by rotating teams rather than individual foster parents. These institutional settings differ from foster-family models in unit size, staffing ratios, and medico-social governance—factors that may shape both mental-health risk and intervention opportunities. Such structural characteristics contribute to the under-representation of French cohorts in international research and justify the present focus on residential care.

Attachment theory provides a robust framework for understanding vulnerability to suicidality. Early caregiver interactions shape internal working models that guide emotion regulation and help-seeking across development [5, 6]. Inconsistent or disrupted caregiving—frequent in child-welfare populations—is associated with insecure or disorganized attachment [7, 8], which predicts anxiety, depression, and suicidal behavior [9]. Distinct attachment dimensions may confer specific risks: attachment anxiety, marked by emotional hyperactivation and fear of abandonment, has been linked to suicidal behavior, whereas attachment avoidance, characterized by emotional disengagement, more often accompanies suicidal ideation [10, 11].

Adverse childhood experiences (ACEs)—including abuse, neglect, and household dysfunction—are pervasive in residential care and exert well-documented effects on socio-emotional development. Beyond psychological sequelae, ACEs alter biological stress regulation and emotional reactivity [12–16], both of which heighten vulnerability to suicidality. Attachment insecurity may further mediate the link between early trauma and suicidal behavior [17, 18], highlighting the importance of relational pathways.

Despite this convergence of risk factors, few studies have examined their combined impact on suicidality in residential care, particularly in the French context. While attachment to multiple figures (mother, father, peers) can influence psychological outcomes, maternal attachment is most consistently associated with internalizing symptoms [9, 19], justifying its focus in the present study. Moreover, although gender differences in suicidality are well established, their underlying mechanisms remain unclear. Existing evidence suggests that girls may be more vulnerable to attachment-related emotional dysregulation, whereas boys may be more affected by cumulative trauma and behavioral dysregulation [4, 20].

### Hypotheses

We expected suicidality to be positively associated with both cumulative ACE exposure and attachment insecurity, and to exhibit gender-differentiated configurations: among girls, a stronger link between maternal alienation, anxiety, and ideation; among boys, a more pronounced association between cumulative trauma, depressive burden, and suicide attempts.

## Methods

### Study design and setting

We conducted a cross-sectional, multicenter observational study between November 2018 and November 2020 in ten residential care facilities in the Bouches-du-Rhône region (France). Facilities were purposively selected to capture a diversity of socio-geographical contexts (urban, peri-urban, rural) and psychosocial trajectories. This study was part of a broader research program on adolescent mental health in child protection systems and was approved by the Aix-Marseille University Ethics Committee (Approval No. 2016-09-11-1). All procedures adhered to national and international ethical standards for research involving minors, including the Declaration of Helsinki (2013). A cross-sectional design was selected to examine concurrent associations between adverse experiences, attachment patterns, and suicidality in a highly mobile population that is difficult to follow longitudinally, as retention is often compromised by placement instability and transitions. Given feasibility constraints in residential care, we targeted ~ 100 participants across 10 facilities, a sample size comparable to prior institutional studies and sufficient to estimate moderate-size odds ratios with acceptable precision (Cohen’s d ≈ 0.50; α = 0.05; power ≈ 0.80).

### Participants and recruitment

Eligible participants were adolescents aged 12–17 years residing in participating facilities during the study period. Inclusion criteria were: (1) sufficient proficiency in French to complete self-report instruments, (2) written informed assent from the adolescent, and (3) written consent from legal guardians or the child welfare judge when required.

Facility directors and care teams were informed about the study and received standardized training prior to data collection. Adolescents were approached individually by trained research assistants unaffiliated with the institutions to minimize social desirability bias. Participation was voluntary and confidential. The final analytic sample comprised 98 adolescents with complete data on the main variables of interest.

### Measures

#### Sociodemographic and contextual variables

A structured questionnaire, developed and validated by an interdisciplinary expert panel (child psychiatry, developmental psychology, social care), collected data on age, gender, schooling level, duration of current placement, number of prior placements, psychiatric history, and family contact. Socioeconomic status (SES) was estimated indirectly based on placement trajectories, educational disruptions, and parental indicators (e.g., unemployment, incomplete schooling), given the limited applicability of conventional SES indices in child welfare populations.

#### Adverse childhood experiences (ACEs)

ACEs were measured using the validated French version of the Adverse Childhood Experiences Questionnaire [21]. The ten binary items assess emotional, physical, and sexual abuse; emotional and physical neglect; and five household dysfunctions (parental separation, mental illness, substance use, domestic violence, incarceration). A cumulative ACE score (range: 0–10) was computed, with higher scores indicating greater adversity. Internal consistency in this sample was satisfactory (Cronbach’s α = 0.74). For descriptive purposes, ACEs were also grouped into abuse-related and household-related domains.

#### Attachment patterns

Attachment security was assessed using the French adaptation of the Inventory of Parent and Peer Attachment (IPPA; α = 0.89) [22], which evaluates perceived attachment to mother, father, and peers across three dimensions (trust, communication, alienation). Each subscale yields a global score, with higher scores reflecting greater security. Although all IPPA subscales (mother, father, peers) were administered, only the maternal composite was retained for multivariate models to align with prior evidence—and our own findings—indicating that maternal attachment is most strongly linked to internalizing symptoms and suicidality. This choice also avoided redundancy among highly correlated sources and preserved events-per-variable in a sample with approximately 33% suicidality. Father and peer subscales are reported descriptively (Table 1) and discussed for clinical interpretation. The maternal subscale (25 items) was standardized (z-scores) for regression analyses, and tertiles were used for descriptive comparisons.

#### Psychiatric symptomatology

Depressive symptoms were assessed with two validated French instruments: the Children’s Depression Inventory (CDI; α = 0.84) [24] and the Adolescent Depression Rating Scale (ADRS) [25]. The CDI served as the primary measure in regression models due to its established clinical thresholds, widespread use, and comparability with prior suicidality research. The ADRS complemented gender comparisons to capture phenomenological variations without introducing collinearity in multivariate analyses.

Anxiety was assessed with the State-Trait Anxiety Inventory (STAI; Form Y; state α = 0.88; trait α = 0.86) [26], distinguishing state anxiety (20 items) from trait anxiety (20 items). All symptom scores were analyzed as continuous variables; validated French cut-offs (e.g., CDI ≥ 19; ADRS ≥ 12) were used in sensitivity analyses to examine clinical thresholds.

#### Suicidality

Suicidal ideation and behaviors were assessed using the Columbia Suicide Severity Rating Scale (C-SSRS) [27], administered by trained interviewers. The C-SSRS distinguishes passive and active ideation, planning, and attempts. In line with Brent et al. [28], a binary outcome was constructed (“no suicidality” vs. “any suicidality”); a three-level ordinal outcome (no ideation, ideation only, attempt) was tested in sensitivity analyses.

### Data quality and missing data handling

All assessments were conducted individually in private settings to ensure confidentiality and optimize validity. Missing data were below 5% for all variables. Little’s missing completely at random (MCAR) test indicated that data were missing completely at random. Multiple imputation using chained equations (MICE, 5 iterations) was applied in sensitivity analyses; results did not differ materially from complete-case analyses, which were retained for primary models.

### Statistical analyses

Analyses were conducted with SPSS v26 (Statistical Package for the Social Sciences) and R v4.3. Descriptive statistics (means, standard deviations, proportions) characterized the sample. Normality of continuous variables was tested using the Shapiro–Wilk test, guiding the use of parametric (t-tests) or non-parametric (Mann–Whitney U) tests; categorical variables were compared with χ² or Fisher’s exact tests. Gender differences in depressive and anxiety symptoms were examined with independent-samples t-tests and effect sizes (Cohen’s d).

Primary analyses used multivariate logistic regression to identify predictors of suicidality (binary outcome). Predictors included gender, age, cumulative ACE score, maternal attachment (IPPA), depressive symptoms (CDI), and trait anxiety (STAI-Trait). All continuous predictors were standardized (z-scores) to facilitate comparison of effect sizes. Model assumptions were checked: all variance inflation factors (VIFs) were < 2, indicating no multicollinearity. Model fit was evaluated using Nagelkerke’s R² and the Hosmer–Lemeshow test.

Given prior evidence of gender-specific pathways to suicidality, stratified logistic regressions were conducted for boys and girls. Interaction terms (gender × attachment) were tested but not retained due to non-significance. Robustness checks included alternative outcome specifications (ordinal suicidality) and substitution of ADRS for CDI in the models; results remained consistent.

Variables such as placement duration, number of prior placements, and psychiatric history were recorded but not included in primary models to limit overfitting relative to the number of events. This analytic structure—combining standardized predictors, multivariate logistic regression, and gender-stratified models—was selected to directly test our hypotheses on cumulative adversity, attachment insecurity, and gender-differentiated pathways to suicidality. Potential residual confounding is addressed in the Limitations, and feasible longitudinal approaches are outlined as future directions.

## Results

### Sample characteristics

Ninety-eight adolescents (54 girls, 44 boys; M = 14.34 years, SD = 2.08) participated. All had been placed in residential care following severe family dysfunction and multiple adverse experiences.

### Adverse childhood experiences (ACEs)

Exposure to ACEs was high, particularly emotional abuse (78%) and physical abuse (65%) (Table 1). Mean ACE scores were higher in girls than boys (M = 5.54 vs. 4.29; *p* = 0.007, d = 0.66). Girls were more likely to report emotional abuse (90.7% vs. 61.9%; *p* = 0.007, d = 0.66), physical abuse (85.2% vs. 61.9%; *p* = 0.021, d = 0.55), and sexual abuse (35.2% vs. 9.5%; *p* = 0.001, d = 0.76).

### Attachment patterns

As pre-specified, only the maternal IPPA subscale entered multivariate models; father and peer scales were examined descriptively (Table 1) to contextualize gender differences.

Attachment insecurity was frequent, with notable gender differences (Table 1). Girls scored higher on maternal alienation (*p* = 0.013, d = 0.53), whereas boys reported greater maternal trust (*p* = 0.023, d = 0.48). Although girls also showed some differences in peer and father attachment dimensions, these variables were not central to the present analyses.

Bivariate correlations indicated that ACE scores were negatively associated with maternal trust (boys: *r* = − 0.52; girls: *r* = − 0.58; all *p* < 0.001) and positively with maternal alienation (boys: *r* = 0.48; girls: *r* = 0.62; all *p* ≤ 0.001) (Table S1).

### Mental health indicators

High rates of emotional distress were observed (Table 1). Trait anxiety was elevated in 45% of participants (scoring above the clinical cut-off), with girls reporting higher trait anxiety (M = 52.13 vs. 43.24; *p* < 0.001, d = 0.77) and higher state anxiety (*p* = 0.020, d = 0.48). While mean CDI scores did not differ by gender (*p* = 0.918), girls had higher ADRS scores (*p* = 0.001, d = 0.69). Across the whole sample, ACE scores correlated positively with depressive symptoms (*r* = 0.49, *p* < 0.001).

### Suicidal ideation and attempts

Overall, 33% of participants reported some form of suicidality (ideation and/or at least one attempt), with no significant gender difference (*p* = 1.00). Suicidality was strongly correlated with ACE scores (boys: *r* = 0.52; girls: *r* = 0.58; all *p* < 0.001) and maternal attachment insecurity (boys: *r* = 0.48; girls: *r* = 0.62; all *p* ≤ 0.001). Maternal alienation was the most salient correlate of suicidal ideation, whereas cumulative ACE exposure was the strongest predictor of suicide attempts.

### Multivariate analysis

Logistic regression models (Table 2) confirmed that cumulative ACE exposure and maternal alienation were the only significant independent predictors of suicidality. In the adjusted logistic regression (Table 2), higher ACE scores were the strongest predictors of suicidality, whereas maternal trust was not (*p* = 0.126). When outcomes were examined separately, suicidal ideation was most strongly predicted by maternal alienation (OR = 2.21, 95% CI [1.38–3.55]; *p* = 0.004), while suicide attempts were best predicted by cumulative ACE exposure (OR = 2.89, 95% CI [1.64–5.12]; *p* < 0.001**).** In adjusted models, cumulative ACEs remained the most robust correlate of suicidality, whereas maternal alienation tracked most closely with ideation. Interpreted clinically, a one-SD increase in ACE load moves an adolescent from low to moderate-to-high risk on routine screening, supporting immediate safety planning and trauma-focused referral. Conversely, elevated maternal alienation flags youths—especially girls—whose presentations center on rumination, interpersonal mistrust, and help-seeking difficulties, for whom attachment-informed interventions (e.g., MBT/ABFT elements) are indicated in addition to standard risk management. This framing clarifies the clinical meaning of the odds ratios and translates statistical findings into actionable guidance for residential-care teams.

### Gender-specific risk profiles

Gender-stratified models suggest two service-relevant pathways: in girls, suicidality is anchored in attachment-based distress (maternal alienation) with high trait anxiety; in boys, it is more tightly coupled with cumulative trauma and depressive load, consistent with more disinhibited risk trajectories. For triage, girls with high alienation benefit from relationally focused care plans (validation, mentalization, family work), while boys with high ACEs and depressive symptoms require trauma-oriented and impulse-control strategies alongside standard safety procedures. These distinct configurations illustrate gender-specific mechanisms of vulnerability. Among girls, attachment-related emotional hyperactivation appears to drive rumination and ideation, whereas among boys, cumulative trauma and depressive burden promote more impulsive, attempt-prone trajectories. These patterns were confirmed in the stratified analyses (Table 2); ACE scores predicted suicidality in both boys (OR = 2.31, 95% CI [1.24–4.02]; *p* = 0.002) and girls (OR = 2.68, 95% CI [1.45–4.91]; *p* < 0.001). For girls, maternal alienation predicted ideation more strongly (OR = 2.01, 95% CI [1.19–3.24]; *p* = 0.005), whereas for boys the association did not reach significance (*p* = 0.081). For boys, suicidality—particularly suicide attempts—was primarily linked to cumulative trauma exposure (with depressive symptoms also contributing), whereas for girls both ACE exposure and maternal attachment insecurity were significant predictors.

Clinically, this differentiation underscores the need for gender-sensitive screening: attachment-based indicators (e.g., maternal alienation, high trait anxiety) should prompt relational interventions for girls, while cumulative ACE load and depressive symptoms should orient trauma-focused and impulse-regulation work for boys.

### Summary of gender differences

Girls’ higher exposure to emotional, physical, and sexual abuse likely contributed to their elevated attachment insecurity and chronic anxiety. While depressive symptoms on the CDI were comparable across genders, ADRS scores revealed greater depressive expression among girls. ACE exposure emerged as the most robust predictor of suicidality across genders, but maternal alienation was particularly salient for girls, emphasizing the importance of gender-sensitive, trauma-informed interventions that address relational dimensions of distress.

These patterns highlight the need for gender-sensitive assessment within residential care, bridging relational and trauma-based approaches.

Girls’ higher exposure to emotional, physical, and sexual abuse likely contributed to their elevated attachment insecurity and chronic anxiety. While depressive symptoms on the CDI were comparable across genders, ADRS scores revealed greater depressive expression among girls. ACE exposure emerged as the most robust predictor of suicidality across genders, but maternal alienation was particularly salient for girls, emphasizing the importance of gender-sensitive, trauma-informed interventions that address relational dimensions of distress.

These findings highlight complementary yet distinct pathways of risk, reinforcing the clinical value of integrating attachment-informed and trauma-focused frameworks in residential care.

**Table 1:** Gender differences in Attachment, trauma Exposure, and psychological symptoms

Variable	Male Mean (SD)	Female Mean (SD)	*p*-value	Effect Size (Cohen’s d)
Adverse Childhood Experiences				
ACE Score	4.29 (2.51)	5.54 (1.92)	*p* = 0.007	Medium
Attachment – Mother				
Total Score	78.29 (14.50)	74.40 (14.21)	*p* = 0.196	Small
Trust	**34.59 (10.66)**	**29.42 (10.76)**	*p* = 0.023	Small
Communication	29.51 (8.44)	26.79 (9.22)	*p* = 0.145	Small
Alienation	**14.20 (7.26)**	**18.19 (7.78)**	*p* = 0.013	Medium
Attachment – Peers				
Total Score	**82.26 (32.10)**	**57.98 (32.11)**	*p* = 0.002	Large
Trust	**34.87 (13.36)**	**24.23 (13.86)**	*p* = 0.002	Large
Communication	**27.19 (12.82)**	**20.18 (11.92)**	*p* = 0.020	Small
Alienation	**20.19 (7.11)**	**13.92 (7.33)**	*p* = 0.001	Large
Attachment – Father				
Total Score	**96.85 (20.52)**	**107.42 (16.11)**	*p* = 0.007	Medium
Trust	43.08 (10.04)	47.00 (9.32)	*p* = 0.056	Small
Communication	**30.75 (8.82)**	**35.83 (5.65)**	*p* = 0.001	Medium
Alienation	23.03 (4.79)	24.60 (4.05)	*p* = 0.092	Small
Depression				
CDI (Children’s Depression Inventory)	26.00 (3.08)	26.08 (3.42)	*p* = 0.918	Negligible
ADRS (Adolescent Depression Rating Scale)	**2.65 (2.33)**	**4.50 (2.98)**	*p* = 0.001	Medium
Anxiety				
STAI-Trait	**43.24 (11.92)**	**52.13 (10.96)**	*p* < 0.001	Large
STAI-State	**36.07 (14.11)**	**42.89 (14.10)**	*p* = 0.020	Medium

*ACE* Adverse Childhood Experiences,* IPPA* Inventory of Parent and Peer Attachment (Mother, Peers, Father subscales),* CDI* Children’s Depression Inventory,* ADRS * Adolescent Depression Rating Scale,* STAI* State-Trait Anxiety Inventory (Form Y: Trait and State). Effect sizes are reported as Cohen’s d: Small (0.20–0.49), Medium (0.50–0.79), Large (≥ 0.80), Negligible (< 0.20). Bold values indicate statistically significant gender differences (*p* < 0.05)

**Table 2:** Multivariate logistic regression analysis of factors associated with suicidality

Variable	Adjusted OR	95% CI	*p*-value	Significance (adjusted model)
ACE Score	**2.45**	**[1.42–4.23]**	*p* < 0.001	Significant (Boys & Girls)
Maternal Alienation	**1.78**	**[1.12–2.82]**	*p* = 0.009	Significant (Girls)
Maternal Trust	0.72	[0.51–1.08]	*p* = 0.126	Not significant
Anxiety (STAI-Trait)	**1.33**	**[1.08–1.76]**	*p* = 0.031	Significant (Girls)
Depression (CDI Score)	**1.41**	**[1.11–1.92]**	*p* = 0.017	Significant (Boys & Girls)

*OR* Odds Ratio,* CI* Confidence Interval,* ACE* Adverse Childhood Experiences,* IPPA* Inventory of Parent and Peer Attachment (maternal subscale: Maternal Alienation, Maternal Trust), * STAI-Trait *State–Trait Anxiety Inventory, Trait form (Form Y-B),* CDI* Children’s Depression Inventory. Odds ratios (ORs) are adjusted for age and sex. OR > 1 indicates increased likelihood of suicidality; OR < 1 indicates a potential protective factor. Bold values indicate statistically significant predictors (*p* < 0.05)

## Discussion

### Attachment and suicidal behaviors

To our knowledge, this is the first study to link maternal alienation to suicidality within a French residential-care cohort, extending prior evidence from foster-care and community samples and underscoring the clinical utility of attachment-informed approaches in institutional settings. A robust literature across developmental psychopathology, attachment theory, and suicidology consistently shows an association between insecure attachment and suicidal behaviors [5, 9]. Secure attachment fosters emotion regulation, self-worth, and help-seeking, whereas attachment disruptions contribute to affective dysregulation, identity fragmentation, and increased vulnerability to suicidal ideation and attempts [7, 19].

Our findings corroborate this evidence, showing a strong link between maternal alienation and suicidality—most notably among girls. The maternal alienation subscale, which captures perceived emotional distance, unavailability, and impaired affective communication, emerged as a significant predictor of suicidal ideation. This aligns with research indicating that insecure or disorganized attachment undermines emotional containment and promotes internalizing symptomatology [23, 29]. In our sample, girls reported higher exposure to emotional abuse and greater maternal alienation, suggesting a convergence of trauma exposure and relational insecurity in shaping their suicidal risk.

This gender-specific pattern mirrors large-scale findings that adolescent girls are more prone to internalizing distress following attachment disruption, while boys more often externalize distress or adopt affective disengagement strategies [30, 31]. In our stratified regression models, suicidal ideation in girls was significantly associated with maternal alienation, whereas in boys, cumulative ACE scores and depressive symptoms were more salient predictors. These divergent pathways underscore the need for gender-sensitive risk formulations.

In the French residential care context, these results are particularly concerning. Although awareness of trauma-related vulnerabilities is increasing, attachment insecurity is rarely assessed explicitly, and interventions often prioritize behavioral stabilization over relational repair [1, 32]. Fragmentation between social, medical, and judicial sectors further impedes integrated, attachment-focused care. Placement instability, a frequent reality for adolescents in care, disrupts continuity of caregiving relationships, fosters mistrust, and reinforces relational disengagement. In such contexts, expressions of alienation are at risk of being misinterpreted as oppositional behavior or mood swings rather than as adaptive responses to repeated relational disruptions.

Emerging clinical and neuroscientific models suggest that attachment-related suicidality may operate through interacting mechanisms, including emotional dysregulation, altered self-representation, and maladaptive interpersonal schemas [33]. One salient pathway involves hypermentalization—the overinterpretation of others’ intentions—observed in trauma-exposed youth with disrupted attachment. Evidence indicates that trauma can impair mentalizing, with dissociation mediating the link between trauma and hypermentalization [34]. In institutional contexts lacking stable adult mirroring, such distortions in self–other understanding may consolidate into mistrust, negative attributional bias, and affective overcontrol, thereby amplifying suicidal vulnerability. Consistent with this, recent evidence in children admitted to emergency departments for suicidal behaviors demonstrated that emotional dysregulation mediates the association between anxious attachment and suicidality, underscoring the central role of regulatory processes in attachment-related risk pathways [35].

Therapeutic approaches such as Attachment-Based Family Therapy (ABFT) and Mentalization-Based Therapy (MBT) offer promising avenues for restoring relational security and enhancing affective modulation. ABFT, while not consistently superior to alternative interventions [36], remains relevant when relational trauma is central to the clinical picture. MBT, by targeting distortions in self–other representation and strengthening mentalizing capacity, may be particularly suited to adolescents in residential care with cumulative adversity and attachment fragmentation [37, 38].

Given the chronicity and complexity of these vulnerabilities, attachment-informed assessment and care planning should be standard practice in institutional child psychiatry. Instruments such as the Child Attachment Interview [39] or trauma-specific attachment screeners could help identify adolescents at elevated relational risk. Staff training in trauma-sensitive relational care is also essential, particularly when embedded within teams to ensure continuity and emotional availability—elements too often absent in large-scale residential environments.

#### Translating profiles into assessment moves

In residential care, we recommend (i) flagging maternal alienation scores in the upper tertile of the IPPA or persistent active suicidal ideation on the C-SSRS as triggers for relationally focused interventions (e.g., MBT-A components, validation training for staff, structured family sessions when feasible); and (ii) flagging ACE scores ≥ 6 and/or clinically significant depressive symptoms as indicators for trauma-oriented care (e.g., TF-CBT elements, affect regulation skills, and close monitoring of impulsivity). Embedding these relational and trauma indicators in admission assessments and weekly clinical reviews can help residential teams move from generic “high-risk” labels toward gender-sensitive, targeted care pathways.

### Neurobiological insights

A substantial body of evidence indicates that early trauma and attachment disruption exert profound neurobiological effects during adolescence, particularly among institutionalized youth. Exposure to ACEs is associated with HPA-axis dysregulation (e.g., flattened diurnal cortisol), a canonical form of biological embedding [40, 41] linked to affective lability and suicidality [42, 43].

At the neural level, trauma-related alterations include amygdala hyperactivation, hippocampal volume reduction, and prefrontal hypoactivation, reflecting impaired regulation of threat detection, emotional arousal, and impulse control [12, 44]. Attachment insecurity may further amplify these vulnerabilities by depriving the child of co-regulatory experiences needed to modulate limbic–prefrontal interactions.

Recent functional-connectivity studies identify dysregulation within the default mode network (DMN)—notably the medial prefrontal and posterior cingulate cortices—as a neural correlate of intrusive rumination, negative self-appraisal, and hopelessness, all strong predictors of suicidality [45–47]. These network alterations may help explain persistent self-referential distress and hopelessness in adolescents in care.

Our findings are consistent with this framework: adolescents with high maternal alienation also showed elevated depressive and anxiety symptoms, particularly among girls, suggesting an interaction between attachment disruption, stress-axis dysregulation, and impaired emotion regulation. While we did not assess cortisol or brain function directly, evidence from related work on borderline traits shows that attachment avoidance and rejection sensitivity predict both cortisol blunting and limbic overactivation [48], supporting the plausibility of similar mechanisms in this sample.

Sex-specific neurodevelopmental trajectories likely modulate these responses. As shown by Roeske et al. (2025), amygdala–prefrontal maturation follows different timelines, with earlier development in females [49]. This may increase girls’ susceptibility to emotion-focused rumination and internalizing distress, while slower prefrontal maturation in boys may foster impulsivity and externalizing behaviors. These developmental asymmetries, converging with our behavioral findings, underline the need to integrate neurobiological and gender-informed perspectives in suicide-prevention frameworks.

Despite these convergences, neurobiologically informed approaches remain rare in French residential care. Ethical and logistical barriers, limited funding, and fragmented services hinder integration of biological knowledge into practice [50]. Somatic and mental-health teams often work in isolation, and caregivers receive little training on the physiological embedding of trauma. This limits implementation of body-based or neuroregulatory interventions—such as trauma-sensitive yoga, neurofeedback, EMDR, or vagal-nerve stimulation—that have demonstrated efficacy in modulating stress physiology and affective regulation [51–53].

Bridging this divide requires a developmental-trauma framework integrating neurobiology, attachment, and environment, with practical steps such as incorporating simple physiological indicators, staff education, and intersectoral protocols toward biologically informed suicide-prevention strategies.

### Gender-specific risk trajectories

Our findings, derived from one of the first French residential-care cohorts examined through a gender-differentiated lens, indicate two distinct, developmentally grounded pathways linking attachment insecurity and early adversity to suicidality in adolescents placed in residential care. Girls reported significantly higher maternal alienation and perceived relational unavailability, patterns strongly associated with internalizing distress. This aligns with evidence that female adolescents are more sensitive to interpersonal stressors, possibly through oxytocin-mediated social-salience mechanisms that amplify the emotional impact of relational rupture and evaluation by others [54]. Interpersonal adversity—such as emotional invalidation, bullying, or rejection—predicts internalizing symptoms and suicidal ideation more strongly in girls, consistent with the relational–affective profile observed in our data [55, 56].

For boys, suicidality was more tightly linked to cumulative trauma exposure—particularly physical abuse and neglect—and to externalizing traits such as impulsivity and irritability. Although inhibitory control was not directly assessed, prior studies suggest that trauma-exposed boys exhibit heightened emotional reactivity and reduced executive control, fostering disinhibited, less premeditated suicidal behaviors [57, 58]. This divergence supports neurodevelopmental stress models in which girls tend to internalize affective distress whereas boys manifest it through behavioral dysregulation and impulsive action [46, 59].

Taken together, these trajectories—characterized by ruminative internalization in girls and dysregulated behavioral control in boys—may constitute distinct mediational routes between ACEs and suicidality. Longitudinal evidence confirms that insecure attachment predicts suicidality via internalizing symptoms in girls and behavioral dysregulation in boys, highlighting gender-specific regulatory vulnerabilities [35].

**Fig. 1:**
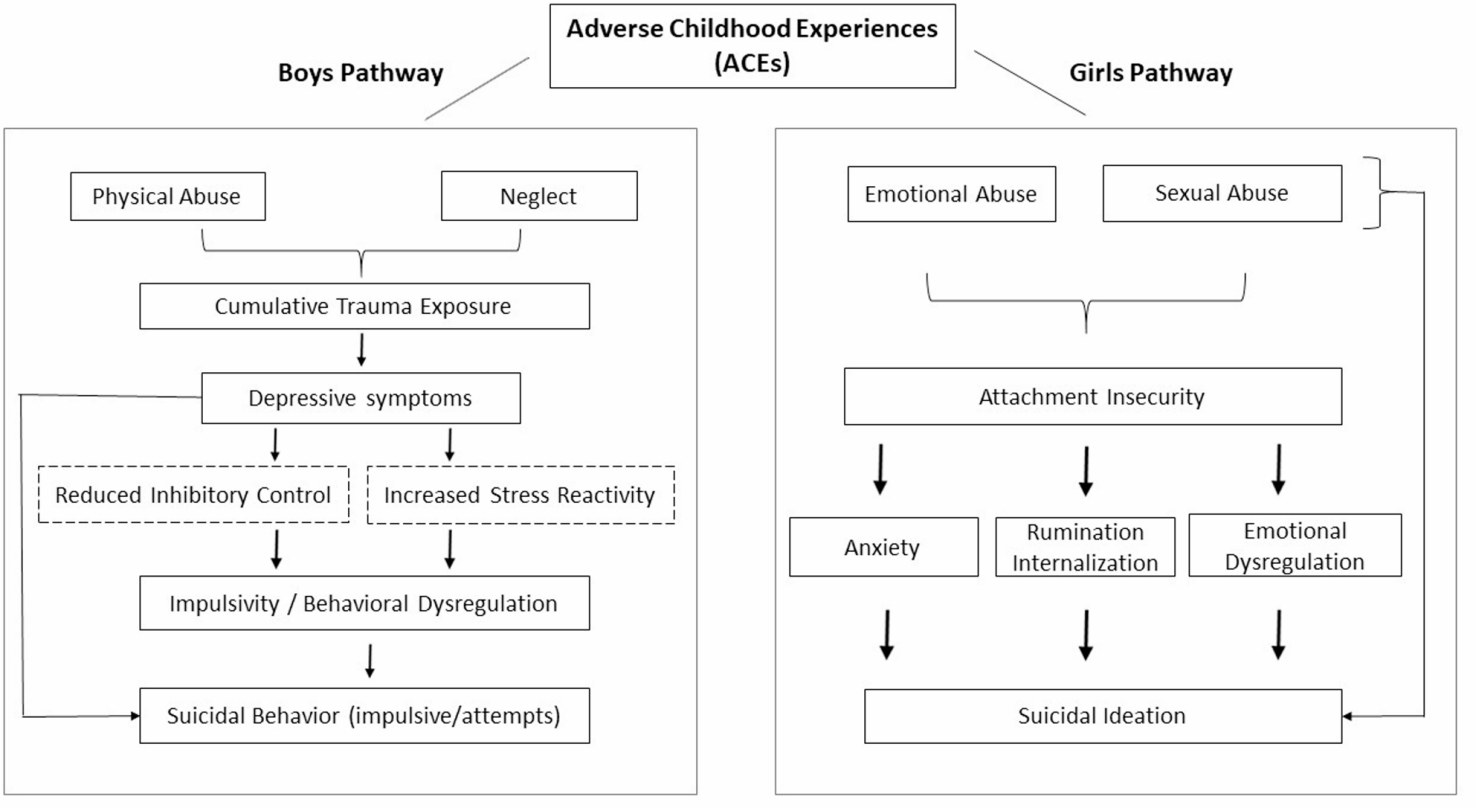
Conceptual model of gender-specific pathways linking adverse childhood experiences (ACEs) to suicide risk in adolescents in residential care. In girls, emotional and sexual abuse were associated with suicidal ideation directly and through attachment insecurity, anxiety, rumination/internalization, and emotional dysregulation. In boys, cumulative exposure to physical abuse and neglect was linked to depressive symptoms and externalizing tendencies, with hypothesized contributions from reduced inhibitory control and increased stress reactivity leading to impulsive suicidal behaviors. Dashed boxes represent hypothesized mechanisms not directly assessed in this study. Arrows indicate proposed direct or indirect relationships, and the order of boxes reflects the suggested sequence of influence

Figure 1 synthesizes these parallel mechanisms, integrating our empirical data with developmental and neurobiological evidence. It illustrates how specific ACE profiles (e.g., relational trauma in girls, cumulative neglect or abuse in boys) interact with attachment insecurity and regulatory vulnerabilities to produce differentiated risk configurations.

From a clinical standpoint, these divergences justify gender-tailored intervention strategies. For girls, approaches that address relational schemas, foster emotional validation, and modulate internal dialogue—such as attachment-informed Cognitive Behavioral Therapy (CBT), Dialectical Behavior Therapy (DBT), or Mentalization-Based Therapy (MBT)—may be particularly beneficial. Boys, in contrast, often require trauma-focused interventions targeting behavioral regulation and impulse control—such as Trauma-Focused Cognitive Behavioral Therapy (TF-CBT), neurofeedback, or somatic-based therapies. Embedding such interventions within institutional care protocols, coupled with staff training to recognize and respond to gendered modes of suffering is essential to improving suicide-prevention outcomes in residential care.

### The worsening mental health of adolescent girls

Our findings are consistent with growing international concern over the disproportionate rise in suicidality and internalizing symptoms among adolescent girls. In the United States, data from the Youth Risk Behavior Surveillance System indicate that the proportion of high school girls seriously considering suicide increased from 19% in 2011 to 30% in 2021—nearly double the rate observed in boys (14.3%) [60]. Similarly, emergency department visits for suspected suicide attempts rose by 51% among females aged 12–17 during early 2021 compared with the same period in 2019 [61].

These trends predate the COVID-19 pandemic. A recent systematic review confirms that adolescent suicide is a multifactorial phenomenon shaped by mental health conditions, family and school environments, and broader social stressors [62]. In the United Kingdom, epidemiological monitoring shows a significant rise in eating disorder rates among adolescents—rising from 0.8% in 2017 to 12.5% in 2023 among 17- to 19-year-olds [63]. This surge in eating disorder prevalence further illustrates the growing psychosocial vulnerability of adolescent girls, which is closely linked to heightened risks of suicidality.

This deterioration appears to result from the convergence of factors in three domains: (1) developmental and neurobiological vulnerabilities—pubertal hormonal changes, particularly within corticolimbic circuits, heighten stress responsivity and emotional volatility, especially in contexts marked by early trauma or chronic stress [49, 59]; (2) relational factors—higher rates of maternal alienation and anxious–depressive profiles among girls point to the role of insecure attachment and interpersonal invalidation in the development of internalizing distress [10, 19]; and (3) sociocultural pressures—appearance-based validation, perfectionistic self-expectations, and upward social comparison, often intensified by digital media, are strongly associated with depressive symptoms, disordered eating, and low self-worth [64–66].

Within residential care settings, discontinuities in caregiving, frequent staff turnover, and limited emotional containment may exacerbate these vulnerabilities. In the French context, repeated placement changes and fragmented support structures can compound the impact of pre-existing emotional vulnerability, leaving adolescent girls with reduced access to stable, validating relationships.

Addressing this crisis requires early, gender-sensitive strategies. Attachment-informed, trauma-focused, and emotion regulation–oriented programs—such as CBT, Acceptance and Commitment Therapy (ACT), and MBT—have shown efficacy in reducing suicidal ideation and depressive symptoms in adolescent girls [67, 68]. Structural measures—including digital literacy education, resilience-building within institutional contexts, and targeted reduction of school-related stressors—are also essential.

In sum, the adolescent girls’ mental health crisis reflects an interplay of neurobiological, relational, and sociocultural forces. These findings directly inform the policy and clinical priorities outlined in Sect. 4.5, underscoring the urgency of developing tailored, context-sensitive approaches within residential care systems.

### Implications for policy and practice

Our findings underscore critical gaps in the mental-health care continuum for adolescents in residential care—particularly in the systematic assessment and treatment of suicidality, attachment disturbances, and trauma-related disorders. In our sample, over one-third of youth reported suicidal ideation or behavior, and attachment insecurity and early adversity were highly prevalent. Despite this burden, existing protocols remain fragmented and inconsistently implemented, rarely tailored to adolescents’ developmental and relational needs [1, 69]. Addressing these shortcomings requires integrated, trauma- and attachment-informed strategies embedded within residential structures.

Action priorities can be organized by feasibility and timeframe:

#### Immediate priorities (next 6 months): strengthening suicide-prevention protocols

Routine risk screening with validated tools such as the C-SSRS should be implemented across all residential units, accompanied by standardized referral pathways and staff training in emergency response [27, 28]. Screening must be gender-responsive: girls with high attachment insecurity should receive relationally focused interventions emphasizing emotional validation and interpersonal safety, whereas boys may benefit from modules targeting impulse control and emotion recognition [57, 59].

#### Medium-term goals (6–24 months): embedding trauma- and attachment-informed care

The convergence of early adversity and insecure attachment calls for interventions that rebuild trust while enhancing regulation capacities. Attachment-informed models such as MBT, DBT, or the Attachment, Regulation and Competency (ARC) framework have shown efficacy in reducing suicidality and improving relational security [38, 70, 71]. Trauma-Informed Care (TIC) principles—viewing dysregulated behaviors as adaptive responses to adversity—should guide daily practice. Key structural levers include staff continuity, emotional containment, and non-punitive caregiving climates.

#### Longer-term reforms (beyond 24 months): integrating mental-health professionals and ensuring systemic coordination

Embedding psychiatrists, psychologists, and trauma-trained therapists within residential teams enables individualized case formulation, rapid crisis response, and longitudinal planning [1, 72]. Ongoing supervision and specialized training in attachment-aware, mentalizing, and affect-regulation approaches can reduce coercive practices and improve relational climates [73, 74]. At the policy level, national frameworks should mandate routine suicide-risk screening, enforce trauma- and attachment-informed standards, and guarantee access to embedded professionals in all placements.

#### Critical transition periods (before and after placement exits): ensuring structured aftercare

Transitions out of care remain high-risk windows often marked by abrupt loss of support. Coordinated aftercare programs—combining peer mentorship, mobile outreach, and sustained psychological follow-up—are essential to reduce relapse risk and foster long-term adjustment [70].

By translating these four evidence-based priorities into graduated institutional and policy actions, child-welfare systems can better meet adolescents’ developmental and psychiatric needs, ultimately reducing suicide risk, and promoting healthier trajectories. The cost of inaction—measured in preventable loss of life and intergenerational trauma—underscores the urgency of systemic reform.

## Limitations and future directions

This study advances understanding of the interplay between trauma exposure, attachment insecurity, and suicidality among adolescents in residential care. While the findings offer clinically and theoretically relevant insights, several methodological and structural limitations must be considered. Addressing these constraints is essential for both interpreting our results and guiding future research aimed at clarifying risk and resilience mechanisms in this high-need population.

### Sample size and representativeness

Although sufficient for exploratory analysis, the relatively small sample size limits the generalizability of our findings. Residential care institutions vary in organizational structure, staffing models, therapeutic frameworks, and institutional climate—factors likely to influence adolescents’ developmental and clinical trajectories. While these contextual variables are specific to the French system (e.g., judicial oversight, staff-to-youth ratios, placement instability), the core associations observed—between cumulative adversity, attachment insecurity, and suicidality, particularly the salience of maternal alienation for girls’ ideation—are consistent with cross-jurisdictional evidence, suggesting that our findings likely extend beyond the French context. Multicenter designs spanning diverse placement types, regions, and demographic profiles are needed to capture this heterogeneity.

Data collection occurred partly in the aftermath of the COVID-19 pandemic, which may have shaped referral patterns and participation rates. Adolescents with the most severe psychiatric symptoms or systemic exclusion may have been underrepresented, potentially leading to an underestimation of suicidality prevalence [75]. Future research should therefore employ inclusive, trauma-informed recruitment strategies in post-pandemic youth mental health studies.

### Cross-sectional design constraints

The cross-sectional design precludes causal inference. While we observed strong associations between attachment insecurity, early adversity, and suicidal behaviors, the temporal direction of these relationships remains unclear. Insecure attachment may precede suicidality, yet suicidal ideation can also emerge as a consequence of cumulative distress and relational fragmentation.

Longitudinal studies are needed to track attachment representations, emotion regulation capacities, and mental health outcomes over time, while considering the moderating effects of placement characteristics—such as caregiver consistency, emotional attunement, and institutional transitions—on these developmental pathways. In practice, such follow-ups are feasible even in mobile populations through mixed-mode strategies (e.g., telephone or online interviews during placement transitions, data linkage across child-protection and health services). Incorporating these pragmatic methods would move the field beyond cross-sectional inference toward developmentally sensitive, longitudinal understanding.

### Measurement limitations and self-report biases

Our reliance on adolescent self-report instruments was necessary to capture subjective experiences, yet such measures are susceptible to recall bias, mood-congruent reporting, and social desirability effects—particularly in sensitive domains like suicidality and maltreatment. Retrospective evaluations of attachment or trauma may be influenced by current emotional distress, complicating causal interpretation.

Future research should incorporate multimodal assessments, combining clinical interviews, behavioral tasks assessing distress tolerance or emotional reactivity, and biological markers of stress regulation (e.g., salivary cortisol, heart rate variability). This approach would enhance validity and provide finer-grained insight into self-regulation processes and trauma sequelae in institutionalized youth [74, 76].

### Resilience processes and intervention research

Our focus on risk factors leaves resilience processes underexplored. Not all adolescents exposed to adversity follow maladaptive trajectories; protective factors such as supportive adult relationships, reflective functioning, and adaptive emotion regulation skills can buffer the psychological impact of trauma and lower suicide risk.

Future studies should systematically investigate these resilience-promoting factors and their developmental plasticity within residential contexts marked by relational discontinuity. Pragmatic trials and implementation studies are also needed to assess the feasibility, effectiveness, and sustainability of evidence-based interventions such as MBT, DBT, and the ARC model in real-world residential settings [38, 70, 71]. Training residential staff in co-regulation and trauma-responsive caregiving should be evaluated for its impact on both youth outcomes and institutional climate.

### Long-term outcomes and structural continuity

Research on residential care remains largely confined to the placement period, yet the transition out of care is a particularly vulnerable juncture. Service discontinuities, emotional instability, and social marginalization during this phase significantly increase the risk of psychiatric relapse and suicidality [69].

Long-term follow-up studies are essential to determine the enduring effects of early adversity, placement histories, and intervention exposure on adult mental health and psychosocial adjustment. Applying implementation science frameworks could help identify sustainable strategies for embedding mental health services within child welfare systems, ensuring continuity of care beyond placement.

In sum, advancing the field will require longitudinal, multi-method, and interdisciplinary approaches that integrate risk and resilience perspectives. Such work is vital for developing context-sensitive, empirically grounded interventions capable of addressing the complex mental health needs of adolescents in care—and for preventing suicide among the most vulnerable youth.

## Conclusion

This study demonstrated that adolescents in residential care face a disproportionate burden of mental health difficulties, with suicidality strongly linked to attachment insecurity and histories of ACEs. Approximately one-third of participants reported suicidal ideation or behavior, confirming suicidality as a major public health issue in institutional settings. The high prevalence of maternal alienation and the marked vulnerability of girls to relational trauma, emotional dysregulation, and self-injurious behaviors underscore the need for targeted, gender-sensitive interventions. Our findings identify two distinct yet complementary risk pathways: cumulative adversity and depressive burden predict suicidality across genders, whereas maternal alienation specifically amplifies ideation among girls.

Integrated relational and trauma-informed frameworks should serve as the foundation of institutional mental health provision. Embedding qualified mental-health professionals within residential teams—alongside systematic staff training in trauma-responsive and attachment-sensitive caregiving—could improve early detection, enhance relational continuity, and foster emotional regulation skills. Routine screening for suicide risk and attachment-related symptomatology across all child welfare placements should become standard practice. In the short term, systematic C-SSRS screening and structured referral protocols are feasible steps; in the medium term, embedding mental-health professionals and implementing trauma-informed training programs should be prioritized.

Beyond immediate implementation, sustainable change will require therapeutic environments that ensure psychological safety, relational stability, and developmental attunement. Future longitudinal research should clarify causal trajectories between ACE exposure, attachment insecurity, and suicidality, while identifying resilience-promoting processes such as mentoring relationships, peer connectedness, and adaptive emotion regulation. Long-term follow-up studies are particularly needed to evaluate the durability of these integrated interventions beyond the placement period.

Integrating gender-sensitive, developmentally attuned suicide prevention strategies into both policy and practice can move child welfare systems toward more equitable and relationally grounded care—offering a pragmatic path to prevent suicide among the most vulnerable youth.

## Supplementary Information

Below is the link to the electronic supplementary material.

Supplementary Material 1. Supplementary material is available online, including Table S1 presenting the bivariate correlations among the main study variables (ACE, attachment dimensions, depressive symptoms, suicidality).

## Data Availability

The anonymized dataset underlying this article is available from the corresponding author upon reasonable request and subject to institutional data protection policies.
