# Supplementary material for: Attachment insecurity, adverse childhood experiences (ACEs), and suicidality in French residential-care adolescents: a gender-differentiated study
Source: Child Adolesc Psychiatry Ment Health. 2025 Dec 15;20:7. doi: 10.1186/s13034-025-01010-3 (PMC12821293; doi:10.1186/s13034-025-01010-3)
Supplement: Supplementary file 1 — Supplementary Material 1. Supplementary material is available online, including Table S1 presenting the bivariate correlations among the main study variables (ACE, attachment dimensions, depressive symptoms, suicidality). [file 13034_2025_1010_MOESM1_ESM.docx]

**Supplementary Material**

**Table S1. Bivariate Correlations Among Key Variables**

| **Pair of variables** | **r (Pearson)** | **p** | **N** |
| --- | --- | --- | --- |
| ACE score – maternal trust | –0.506 | p < 0.001 | 94 |
| ACE score – maternal communication | –0.415 | p < 0.001 | 94 |
| ACE score – maternal alienation | 0.534 | p < 0.001 | 94 |
| ACE score – peer trust | –0.492 | p < 0.001 | 71 |
| ACE score – peer communication | –0.439 | p < 0.001 | 71 |
| ACE score – peer alienation | –0.473 | p < 0.001 | 70 |
| ACE score – depressive symptoms (CDI) | 0.490 | p < 0.001 | 98 |
| ACE score – suicidality (C-SSRS) | 0.580 | p < 0.001 | 98 |

**Note.** Only correlations involving the ACE composite score are tabulated here for brevity; correlations among other variables (e.g., maternal trust ↔ suicidality) can be computed similarly. All reported correlations are two-tailed. Effect sizes are considered small for |r| = 0.10, medium for |r| = 0.30, and large for |r| ≥ 0.50. Sample sizes vary due to missing data on some subscales; pairwise deletion was used for each correlation.
